# A New Adaptive Method for the Extraction of Steel Design Structures from an Integrated Point Cloud

**DOI:** 10.3390/s21103416

**Published:** 2021-05-14

**Authors:** Pawel Burdziakowski, Angelika Zakrzewska

**Affiliations:** 1 Department of Geodesy, Faculty of Civil and Environmental Engineering, Gdansk University of Technology, Narutowicza 11-12, 80-233 Gdansk, Poland; 2 Geopartner Spolka Z Ograniczoną Odpowiedzialnością Spolka Komandytowa, ul. Rakoczego 31, 80-171 Gdańsk, Poland; zakrzewska.ang@gmail.com

**Keywords:** photogrammetry, TLS, UAV, steel structure, monitoring, integration, fusion

## Abstract

The continuous and intensive development of measurement technologies for reality modelling with appropriate data processing algorithms is currently being observed. The most popular methods include remote sensing techniques based on reflected-light digital cameras, and on active methods in which the device emits a beam. This research paper presents the process of data integration from terrestrial laser scanning (TLS) and image data from an unmanned aerial vehicle (UAV) that was aimed at the spatial mapping of a complicated steel structure, and a new automatic structure extraction method. We proposed an innovative method to minimize the data size and automatically extract a set of points (in the form of structural elements) that is vital from the perspective of engineering and comparative analyses. The outcome of the research was a complete technology for the acquisition of precise information with regard to complex and high steel structures. The developed technology includes such elements as a data integration method, a redundant data elimination method, integrated photogrammetric data filtration and a new adaptive method of structure edge extraction. In order to extract significant geometric structures, a new automatic and adaptive algorithm for edge extraction from a random point cloud was developed and presented herein. The proposed algorithm was tested using real measurement data. The developed algorithm is able to realistically reduce the amount of redundant data and correctly extract stable edges representing the geometric structures of a studied object without losing important data and information. The new algorithm automatically self-adapts to the received data. It does not require any pre-setting or initial parameters. The detection threshold is also adaptively selected based on the acquired data.

## 1. Introduction

Today, measurement technologies for spatial modelling data are under continuous and vigorous enhancement. The most popular methods include photogrammetric techniques based on digital visible light cameras and laser scanning. The development of these sensors drives engineers and scientists to come up with newer measurement methods and associated applications. More and more of the above find their application in civil engineering [1,2,3], environmental engineering [4,5,6], construction [7,8,9,10] or architecture [11,12], thus intensively stimulating the further progress of these technologies. 

When using the aforementioned photogrammetric techniques, the differences arising from the various types of used sensors should be taken into account. On the one hand, we are dealing with a passive sensor: a photo camera, the images from which constitute a basis for further geometric studies; on the other hand, a laser scanner that collects information on the surrounding terrain via an active sensor, most usually in the red band. Various sensors and different methods for the acquisition of spatial information result in such data also being different. Both technologies have their pros and cons, which are described in more detail in [13]. Their simultaneous use cross-eliminates the restrictions of both sensors. Information from two or more sensory sources can be fused or integrated, which supports the modelling process and minimizes the modelling issues arising from the physics of a given sensor.

Quite often, the data acquired using an unmanned aerial vehicle (UAV) constitute a perfect complement to the terrestrial laser scanning (TLS) data. Therefore, these techniques can be deemed complementary. 

In general, as indicated by a review of the source literature below and the nature of both technologies, it should be concluded that TLS information is used to generate a true geometric model (quantitative data), while visible light camera or multispectral camera data additionally provide qualitative and quantitative data. The source literature already contains multiple methods for fusing UAV and TLS data, and the number of their applications is constantly growing. The authors of [14,15,16] developed an improved method for assessing landslide risk based on a generated 3D surface model. UAV photos were used within the research to assess slope-forming rock cracks. The synergistic use of photogrammetric products and their fusion is often the case in the assessment of landslide risks, which is demonstrated by [17,18]. The authors of [19] concluded that the method for acquiring photos from a UAV is characterized by higher accuracy in modelling key forest properties during its regeneration phase. In their publication [20], the authors compared data acquired via stationary laser scanning and data from a scanner on-board a UAV. This is a concept created by the Austrian company Riegl. UAV Laser Scanning (ULS) proved to be more efficient, faster and more accurate in the case of forest areas than the stationary method that is recognized as the reference in this study. As also noted, airborne laser scanning (ALS) provided lower-density clouds that, in the case of forest areas, failed to guarantee data—an aspect that is vital in terms of this object. The combination of image and laser scan data is widely used in forestry. In the case of [21,22], data from two different sources significantly improves the elaboration quality and the ultimate initial point accuracy. 

Low-altitude photogrammetry (or UAV photogrammetry) was found to be excellent for an accurate analysis of coastline and littoral areas [23,24,25]. The study [26] thoroughly assessed the accuracy of the applied digital surface model (DSM) that was aimed at detecting changes in a coastal area. The authors of [25] also presented a filtration method involving UAV data that was intended to enhance the matching of coastal area models. The publication [27] comprehensively described a method of fusing sensory data for coastal protection systems.

Salach et al., in [28], thoroughly analysed the accuracies achieved owing to UAV Laser Scanning (ULS) and UAV-photogrammetry, in which case it was concluded that the Digital Terrain Model (DTM) generated by ULS was significantly more accurate and enabled the elimination of inaccuracies related to terrain vegetation. The authors indicate that laser technology had clear advantages over photogrammetric models in situations where vegetation can be a problem during terrain surface reconstruction. In contrast, in the case of terrains not covered by vegetation, UAV photogrammetry enables the achievement of surface model determination accuracy from 1 cm [29]. 

Information on the natural environment can also be enhanced owing to use of multispectral sensors and the integration of these with spatial data. Salehi et al., in [30], reviewed a methodology for integrating multispectral camera and scanning laser data for the evaluation of sea cliffs in the Arctic region. Bujakowski et al., in [31], stated that the data from ALS and multispectral photography constituted grounds for the assessment of embankment stability. 

Very good results are also achieved by combining scans and a photogrammetric model when studying engineering structures. Such data provide increased amounts of information and enable the precise stocktaking of cultural heritage structures [32]. Furthermore, owing to numerical and spatial models, the damage and degradation of cultural structures are assessable [33]. The research [34] was conducted from a similar perspective, in which laser scans were used to develop orthoimages to be used as a base to detect structural cracking. Moreover, scans and images can be integrated in order to obtain even more information, which in the case of structural assessment is an innovative method, and was described for the first time in [35]. 

The analysis of the geometry obtained from a point cloud was described in [35], which, just like the previously presented publications, focused on converting the cloud into orthoimages, then subjected these to analyses (e.g., edge detection). It should be noted that these methods develop a two-dimensional image representation (orthoimage), which is then assessed. 

A very interesting publication [36] discussed the possible use of photogrammetric data for the supplementation of airborne laser scanning (ALS) data. Airborne scanning is characterized by the generation of a relatively low density of points; hence, high-resolution photos are perfect to complement the missing data. It is worth mentioning that the authors of [37] suggested reconstructing characteristic geometric structures (building roof outlines in this case) using integrated spatial data. Extracting only vital geometric structures enables the achievement of a significant data volume reduction. 

It should be recognized that point clouds and high-resolution imagery carry large amounts of information. Their magnitude, therefore, can be limited only to what is essential, e.g., by isolating vital geometric structures. This issue was addressed by such elaborations as [38], in which Serna et al. used huge point clouds to extract only the objects that were important from the modelling perspective (building facades in this case). Xie et al. in [38] also presented an urban area building shape extraction method. In addition, they discussed methods of filtering and preparing the data for analyses.

In the case of the stocktaking of engineering structures, high-accuracy spacing mapping for the purposes of reconstruction or comparison is a very important issue. Publications [39,40,41,42] have thoroughly described the comprehensive use of measuring devices in order to improve the accuracy. What is more, they list and develop appropriate algorithms for the evaluation of structural performance. 

Very often, the mapping accuracy in such analyses must be at a level of 1 mm; however, in the case of the object described in this on-going article (a complex steel structure), its dynamic operation and erection precision must fall within a tolerance of 1 cm. It is quite complicated to achieve such a result; therefore, in our article, we propose an innovative method for combining data in order to achieve the required outcome.

Integrated spatial data has a very large number of points. The integration of TLS and UAV clouds results in a number of points that commonly exceeds several million. In most engineering applications, such dense point clouds are not required, and only some characteristic elements of the structure—such as its edges—are analyzed [43,44,45,46]. Additionally, as in the presented case, the constructed object is compared with the design data in CAD (Computer Aided Design) software. Such CAD projects contain mainly lines, representing the edges of the object and its elements. Therefore, it seems reasonable to implement a method to extract only such characteristic features of an engineering structure from a fully integrated point cloud.

As the literature analysis indicates, edge extraction techniques from point clouds can be divided into methods using rouboust statistics [47,48,49,50], surface segmentation [51,52], line segmentation [53], region growing methods [54,55,56,57] and neural methods [58,59]. The application of the methods ranges widely, including robotics [60], reverse engineering [61,62], manufacturing industries [63,64,65] and cartography [46]. One feature common to the above-mentioned methods is the sensitivity to the noise present in the point cloud. Due to the fact that point clouds derived from real measurements of engineering objects generally have a large amount of noise, the selected method should have some noise robustness, while the process of preparing the cloud for analysis should also take this fact into account. 

This study integrated TLS data with UAV image data in order to reconstruct a complex spatial steel structure and then minimize the volume of data and automatically extract vital structural elements from the perspective of engineering analyses. The outcome of the research was the development of a technology for the acquisition of precise complex spatial information related to a high steel structure. This contains such elements as a method for integrating data and using it to extract vital structures, as well as methods for eliminating redundant data and for filtering integrated photogrammetric data. Ultimately, the subsequently applied structure extraction algorithms isolate structural elements that can be easily compared with best steel structure design practices, and consequently evaluate them in terms of execution. In the work, the developed final product, owing to the minimization of the volume of spatial information and the isolation of vital elements, was compared with a theoretical 3D model of the structure.

This study presents the following new solutions in the field of spatial measurements and data analysis:

- The development and presentation of a complete integration technology for spatial data generated from two sensory measurements: data from TLS and that from airborne photogrammetry obtained through UAV flights was integrated.
- The comparative analysis of the developed models and the accuracy analysis of the integration process.
- The development and testing of a new adaptive and automatic algorithm for the extraction of the edges of geometric structures from point clouds.
- A new algorithm used to develop a reduced spatial model of a building’s steel structure.

Within this context, the paper has been organized as follows: the first section is the Introduction, which presents the motivation and background of this study; the second section, Materials and Methods, describes the tools and methods used to process the data, and presents the developed extraction algorithm. The third section discusses the results and quality obtained. The paper ends with a section entitled “Conclusions”, which summarizes the most important aspects of the study.

## 2. Materials and Methods 

### 2.1. Object History and Description

**Figure 1:**
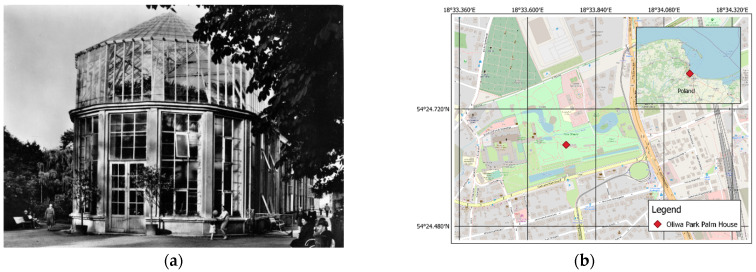
(**a**) Oliwa Park Palm House, 1972–1978 (photo credit: Andrzej Zborski). (**b**) Site location (WGS-84).

The subject matter of the study was the Palm House of the Oliwa Park in Gdansk (Figure 1), constructed in the second half of the 18th century. It is located within the Adam Mickiewicz Park, which occupies an area of almost 10 ha. This park used to be a monastery garden established by the Cistercians and inspired by the French garden ark of the Baroque. The palm house located therein acted as a winter garden housing exotic plants [66].

**Figure 2:**
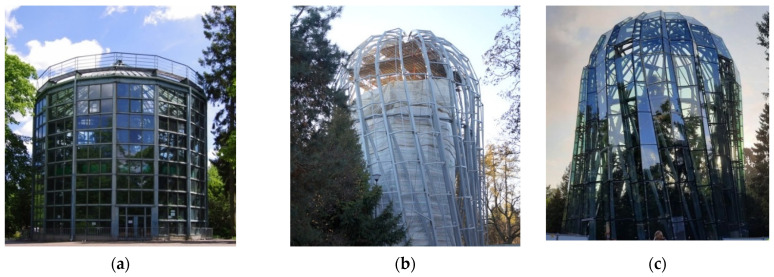
(**a**) Palm house prior to its reconstruction, 2017; (**b**) during the reconstruction, 2018, measurement and construction process inspection period; (**c**) the glazed and commissioned building [66] (Reproduced with permission from Dyrekcja Rozbudowy Miasta Gdanska).

The inside of the building houses palms, cacti, aloe, philodendrons and banana trees in near-natural conditions. The palm house, as an element of a post-Cistercian complex, was entered into the register of monuments in 1971. The date plant therein is 180 years old, and it is the only such object in Poland. Prior to its renovation, the facility consisted of an eastern, single-story brick building. Its cylindrical body was constructed in 1954. The dome—the southern section and parts of the western section of which are glazed—was 15 m high (Figure 2). 

The palm house structure was demolished in September 2017 in order to replace it with a taller building that would incorporate the height of the date tree, which was distorting the roof structure as of 2013. The new structure is cylindrical, and has a glass rotunda with a height of 24 m and a width of 17 m. The volume of the building is 4.4 thousand m^3^. Of note, 1400 supporting points were installed on the steel structure, each of which was individually fitted. 

### 2.2. Process Description

**Figure 3:**
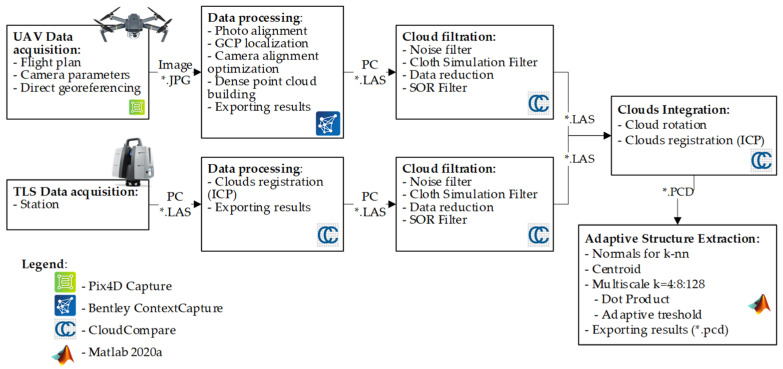
Data processing algorithm (PC: point cloud; *.JPG: JPG file format; *.LAS: LAS file format).

A work methodology and algorithm were developed in order to process the measurement data and isolate the geometric structures of the studied building (Figure 3). The individual stages of the algorithm below are thoroughly discussed in the further sections of this research paper.

### 2.3. Data Acquisition

**Figure 4:**
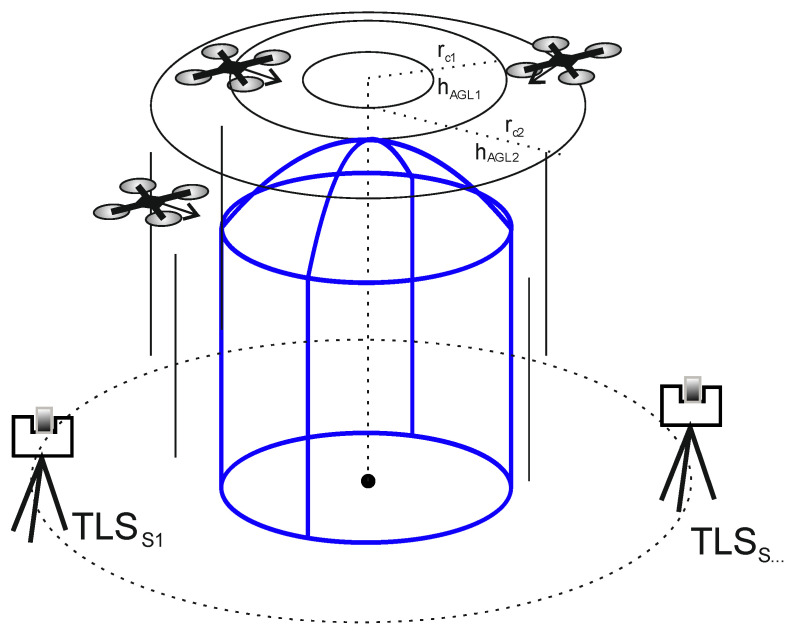
TLS and UAV data acquisition diagram for high structures.

The data acquisition process was conducted using terrestrial laser scanning and UAV flight image acquisition. The fact that the upper section of the facility was unavailable to a laser scanner necessitated the use of a UAV with a non-metric camera. Figure 4 shows a graphical data acquisition diagram. TLS stations were uniformly distributed around the building. In this case, the laser scanner was based on 17 stations. The distance of the scanner from the measurement object was determined experimentally, and is a certain compromise between the available space and the theoretical density of the measurement points. The essence of the determination of the distance is to choose such a distance from the structure as to obtain a combined coverage with the TLS data for the bottom part and the UAV data for the upper. 

Each UAV flight followed a circle with several different radii ($r_{c1}$, $r_{c2}$) and at respectively different altitudes ($h_{AGL1}$, $h_{AGL2}$). Additionally, several vertical flights were conducted in order to photograph the structure below the dome. Figure 4 contains a diagram with circular flight trajectories marked in red, which constitute the theoretical minimum. It also contains the vertical flight trajectories that are advocated for the scanning of such structures. In practice, flying over numerous concentric radii is recommended. The objective of such a flight plan is to maximize the overlapping of the photos and to multiply the projecting rays for a selected area. Two independent flights were applied in the case in question. The first one covered seven concentric trajectories over the structure in a clockwise direction, while the second included nine counter-clockwise concentric trajectories. Some of the trajectories were executed automatically using the available UAV flight automation functions, whereas those at a short distance over the building were conducted manually, as manual flight control over such a structure improves the air operation safety.

#### 2.3.1. UAV Photogrammetry: Initial Data Processing

**Figure 5:**
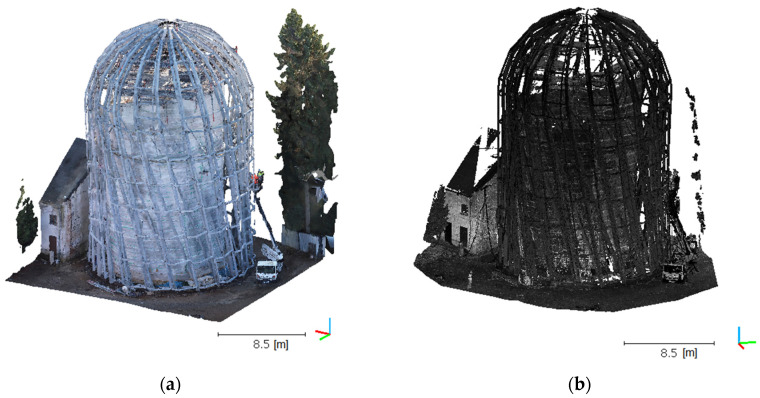
Developed primary point clouds for UAV (**a**) and TLS (**b**).

The photogrammetric flight was conducted using a DJI Mavic Pro (Shenzhen DJI Sciences and Technologies Ltd., Shenzhen, China) UAV. Such an UAV is representative of the commercially available aerial vehicles designed and intended primarily for recreational flying. It was equipped with an integrated non-metric camera. A total of 1180 photos bearing metadata with the current UAV position were taken during the two flights. The data was saved in EXIF (Exchangeable Image File Format). The results were processed using commercial Bentley ContextCapture (Bentley Systems Inc., Exton, PA, USA) software (Table 1). The result of the processing was exported to a point cloud in a *.las format (Figure 5). The UAV image data were processed using the direct georeferencing method, which means that each image contained location data recorded using an UAV on-board global navigation satellite system. Due to the height of the structure and its design, ground control points could not be placed on the object. With the direct georeferencing method, the object was modeled according to its actual scale.

#### 2.3.2. TLS Initial Data Processing

The laser scanning was conducted in a continuous mode using a Leica P30 (Leica Geosystems AG: Part of Hexagon, Sankt Gallen, Switzerland) scanner (Table 2). The measurement stations (17 in total) were placed on the ground, evenly around the structure. The measurements were taken using the option of recording up to a million points per second.

The robust estimation method and the well-known ICP (Iterative Closest Points) algorithm were used in order to align the images from the individual scans. This method aims to appropriately filter the points in order to determine automatic reference points on the station’s point cloud, and then combine them relative to the subsequent stations. The method utilizes an algorithm described in [16], in which the author aligned stationary stations relative to airborne ones using the least squares method. Another interesting modification of the ICP algorithm is presented here [67]. The record alignment results are shown in Table 3, as appropriate translations of PX, PU, PZ (scan shift over individual axes), as well as Roll, Pitch and Yaw (inter-rotation of the stations). PKT is the automatically computed number of reference points that must be taken into account in the calculations. The processing result in the form of a point cloud was exported to the *.las format. 

### 2.4. Point Cloud Filtration

The point clouds generated within the previous stage have a certain amount of redundant data that is irrelevant from the point of view of the extracted structures, and a certain amount of noise and random data (Figure 5). For this reason, the developed point clouds were pre-filtered. As demonstrated in [68], cloud pre-filtration is very important and enables the isolation of vital infrastructure elements. Pre-filtration was also applied in [69]. Pre-filtration consists of four stages: noise filtering, cloth simulation filtering [70] (CSF), data reduction and statistical outlier removal (SOR) filtering. The same stages were applied for each of the acquired point clouds and are recommended prior to the cloud integration stage.

**Figure 6:**
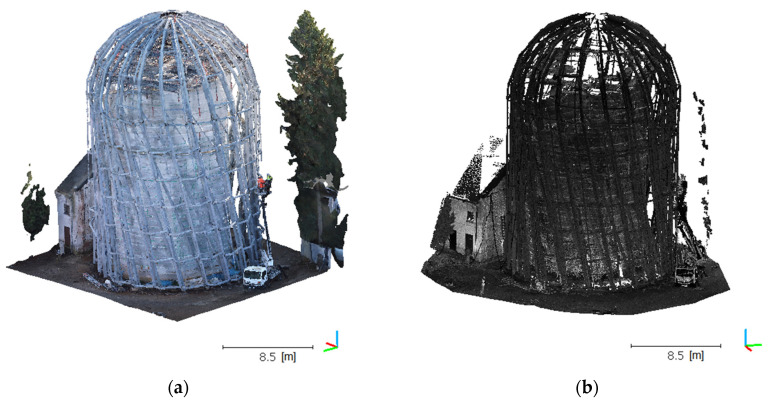
Point clouds after noise filtration for UAV (**a**) and TLS (**b**).

A Surface Distance-Based Filter [70] was applied in the case of the point cloud acquired using an UAV. This filter eliminates outliers (considered noise) that do not fall within a defined distance from the local surface, as determined inside a kernel window defined by a search radius. In this way, it is possible to eliminate noise, i.e., points beyond the minimum distance $(D_{min}$), which are defined as:

(1) $$D_{min}=\underline{sd_{k}}+n\sigma \left[-\right]$$

where $\underline{sd_{k}}$ means the mean distance from the local surface determined by k of the adjacent points around an indicated central point, n is a user-defined coefficient and usually takes the value of 1–3, and σ is the standard deviation of the distance from the flat surface. It should be noted that setting overly aggressive parameters for this method can lead to excessive point cloud filtration. This process can be iterative in order to avoid this. Such filtration also tends to remove rounded surfaces and edges. In the case in question, the $\underline{sd_{k}}$ value was set at 0.006474 m, while n adopted the value of 1. This operation enabled the elimination of 90,290,876 outliers. After this stage, the number of points in the UAV cloud was reduced to 92,214,210 (Table 4) (Figure 6a).

A neighbourhood distance filter was used in the case of the TLS cloud in order to eliminate outliers. These filter k studies define the neighbours of a point for each of the points within a tested cloud; points with a distance higher than the sum of the mean distance and standard deviation values are classified as outliers. This can be expressed as follows:

(2) $$D_{min}=\underline{d_{k}}+n\sigma \left[-\right]$$

where $\underline{d_{k}}$ is the mean distance k of points adjacent to the measured point (centre) and n is a user-defined coefficient that usually takes the value of 1–3. The elimination of the outliers for the studied cases was conducted for k=6 neighbours and n=1. The use of the algorithm resulted in the removal of 42,889,276 points deemed noise from the TLS cloud. The number of points after this operation was 60,791,121 (Table 4) (Figure 6b).

The next stage of the pre-filtration is the removal of the points representing the Earth’s surface and other objects located in the vicinity of the studied structure. The cloth simulation filter (CSF) followed by the manual elimination of small ambient objects was applied for this purpose. The CSF technique [70] enables the segmentation of point clouds and their division into points representing the ground and other elements placed on it.

Cloth simulation is a collision detection algorithm. These are used in computer graphics and computer simulations in order to find movement restrictions in 2D and 3D scenes. In general, a collision detection algorithm answers the following question: is moving any object in a given direction possible or are there obstacles in its path, i.e., other moving or stationary objects? Collisions between various fragments of the same object should also be detected as part of the cloth simulation. Certain modifications were introduced in order for this algorithm to be used for point cloud filtering. Collisions are detected by comparing the heights of the simulated cloth particle and the terrain. As soon as a particle reaches ground level, it is immobilized. The simulation provides an approximation of the real terrain, and then the distances between original cloud points and the simulated particles are calculated using an algorithm for the calculation of the distances between clouds. Points with distances smaller than a defined distance threshold are classified as ground, while the others constitute measurement (terrain) objects.

The practical implementation of the CSF algorithm requires the definition of three parameters. The first is the cloth resolution, which relates to the grid size. The next value concerns the number of iterations. Usually, 500 iterations are sufficient. The last parameter is the classification threshold, which defines the distance between points and the simulated terrain. In order to filter both clouds (UAV and TLS), we assumed the following parameter values: grid size 2500 iterations, and 0.5 for the classification threshold. This eliminated the points classified as the ground surface, and the total number of points in both clouds was once again reduced (Table 4).

**Figure 7:**
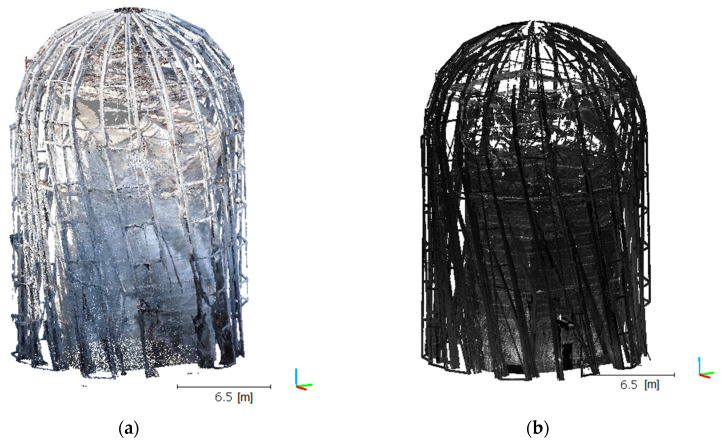
Point clouds after removing the ground and accompanying objects for UAV (**a**) and TLS (**b**).

After eliminating the ground surface, objects located in the vicinity of the studied structure were removed manually. They included a bucket truck and elements of technical infrastructure that the analysis did not cover. After this operation, the UAV and TLS point clouds were deemed fully cleaned and ready for another density balancing operation (Figure 7).

The next step in preparing the clouds for integration is balancing their density. A cloud of higher density should be reduced by a determined density reduction factor ($R_{D}$). For the purposes of this study, the reduction factor was defined as

(3) $$R_{D}=\frac{100}{\left(D_{PC\mathrm{HI}\;}/D_{PCLOW}\right)\;}$$

where $D_{PCHI}$ and $D_{PCLOW}$ are the mean densities for the clouds with higher and lower density, respectively. The mean cloud density ($D_{PC}$) was defined as the product of the sum of the mean surface density ($D_{i}$) of the cloud for k-neighbours of the studied point, with a radius of *r*, and the total number of points in this cloud should be

(4) $$D_{PC}=\frac{1}{n_{T}}\;\overset{n_{T}}{\underset{i=1}{\sum }}D_{i}$$

(5) $$D_{i}=\frac{n_{i}}{\pi r^{2}}$$

where $D_{i}$ is the cloud surface density (points/m^2^), $n_{i}$ is the number of points adjacent to the studied point *i*, *r*–radius (m), $n_{T}$ is the total number of points in a cloud. 

Using the expressions above, the mean density and the UAV cloud reduction factor were calculated for both data sets: UAV and TLS. Consequently, the mean UAV data density amounted to 4973.59 (points/m^2^), the mean TLS data density was 1624.03 (points/m^2^), and the reduction factor was 32.65%. As a result, the number of points in the UAV cloud was reduced and the densities of both clouds were balanced. The number of points in the UAV cloud after this operation was 24,160,311 (Table 4).

The ultimate stage in preparing the data for integration is filtration based on a statistical filter [71,72]. This filter is based on the assumption that an outlier is considered to be a point located further than the adopted threshold, defined as the mean standard deviation distribution for all of the k-neighbours of each cloud point. As such, if we let point $m_{i}$ described with coordinates $x_{i},\;y_{i},\;z_{i}$ within space ${\mathbb{R}}^{3}$ belong to point cloud M with a total number of points $M_{p}$, then

(6) $$M=\left\{m_{i}\right\},i=1,\ldots ,M_{p},m_{i}=x_{i},y_{i},z_{i}$$

And let $m_{q}$ mean a studied point, such that $m_{q}\in M_{i}$, and $m^{n}$ means its neighbouring point wherein $m^{n}\in M_{i}$, then the closest neighbourhood $M^{n}$ k of points adjacent to the studied point $m_{q}$, such that $M^{n}=\left\{m_{1}^{n},\ldots ,m_{k}^{n}\right\}$, satisfies the condition:

(7) $$\sqrt[{p}]{\overset{k}{\underset{1}{\sum }}{\left|m_{k}^{n}-m_{q}\right|}^{p}}\leq d_{m}$$

where $d_{m}$ is the maximum adopted distance between the studied point, and $m_{k}^{n}\in M^{n}$, as well as p≥1 (here adopted p=2).

In consequence, if the mean distance around point $m_{q}$ relative to all points k in its vicinity is

(8) $$d_{i}=\frac{1}{k}\overset{k}{\underset{1}{\sum }}\sqrt{{\left(m_{k}^{n}-m_{q}\right)}^{2}}$$

And for all points $m_{i}$, the mean value of $d_{i}$ is

(9) $$\mu =\sum_{i}^{M_{p}}\frac{d_{i}}{M_{p}}$$

The standard deviation for the studied set M can be defined as

(10) $$\xi =\sqrt{\frac{1}{M_{p}}\;\sum_{i}^{M_{p}}{\left(d_{i}-\mu \right)}^{2}}$$

Thus, the resultant point cloud $M_{o}$, without outliers relative to the mean point will be defined as follows:

(11) $$M_{o}=\left\{m_{q}\in M|\left(\mu -\alpha \xi \right)\leq d_{i}\leq \left(\mu +\alpha \xi \right)\right\}$$

where *α* is an experimentally determined multiplier for a given point cloud.

**Figure 8:**
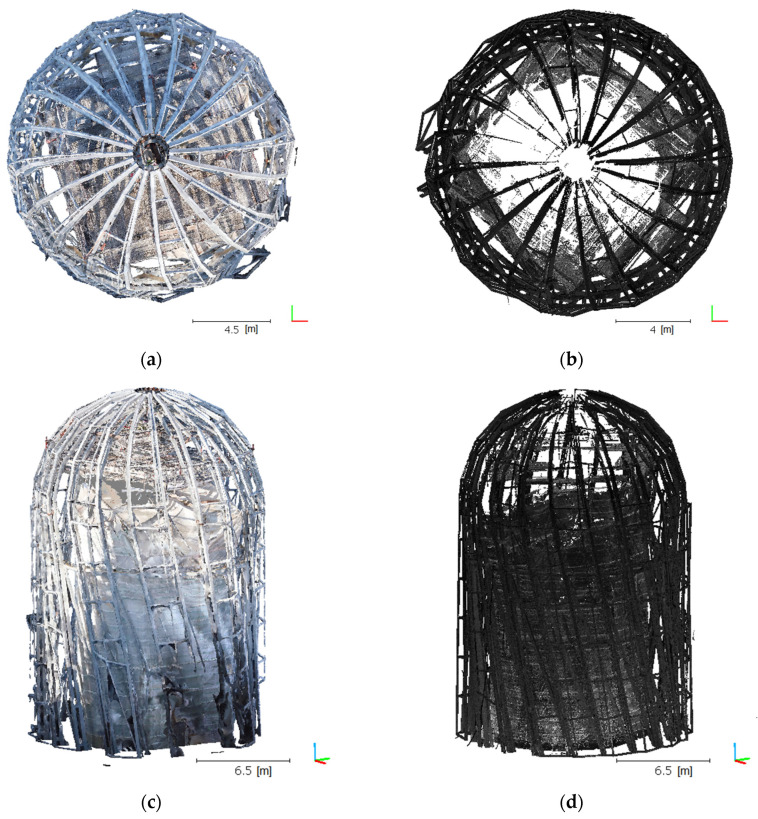
Post-filtration point clouds for UAV: (**a**) top view, (**c**) side view and TLS, (**b**) top view, and (**d**) side view.

The aforementioned statistical filter was applied only once for any given cloud. In the case of the UAV data, we adopted *k* = 6 and *α* = 1, and *k* = 8 and *α* = 4 for TLS, which enabled the ultimate elimination of the outliers (Table 4). Results in the form of a cloud image are shown in Figure 8, which indicates that a UAV cloud clearly better maps the geometry in the upper part of the object, especially near the peak rosette. The TLS cloud does not exhibit complete object geometry in this section. TLS cloud noise and irrelevant data were removed, which revealed the shortcomings of this model. This was a predictable situation because the scanner was positioned in the bottom object section, such that it was not physically possible to fully map the object in this area.

### 2.5. Point Cloud Integration

Point cloud integration is the final process in preparing the data for geometric structure extraction. In this case, the integration will successively use 4-Point Congruent Sets (4PCS) [73] and Iterative Closest Point [74,75,76] (ICP) algorithms. Integration, in fact, involves, in this case, the determination of elementary rotation matrices $R_{X}\left(\theta \right),R_{Y}\left(\theta \right),R_{Z}\left(\theta \right)$ and the 3D coordinates of the translation vector ${\vec{T}}_{X},{\vec{T}}_{Y},{\vec{T}}_{Z}$. This procedure is often encountered when undertaking similar tasks [77].

Cloud integration is conducted in two stages. The first stage is coarse matching and the next is precise matching. As described above, the data was significantly filtered and denoised. However, it should be noted that the data sources differ, and that the modelled surfaces of the structural elements have a slightly different shape depending on the data source. TLS cloud objects have sharp and clear shapes. Metal section cross-sections are very sharp; however, due to occlusions, some of the closed sections only have one part modelled (usually, the outer) that is directly illuminated by the laser beam. The model based on UAV has slightly more rounded section edges. The cross-section of the metal sections is geometrically correct, and has rounded and smoother edges. This is directly related to the characteristics and accuracy of photogrammetric modelling. The phenomenon of occlusion did not have such a significant impact on the data volume, and most sections were completely modelled. Minimizing occlusion results directly from the number of stations taking the photographs. In practice, these were hundreds of positions, whereas in the case of TLS, there were 18 stations. It follows that the 4PCS algorithm, as preliminary matching, will fit perfectly in this case. This was also demonstrated in [53]. As a consequence, the outcome of preliminary matching involving a cloud balanced with the 4PCS algorithm was the following values of the rotation matrix R and transformation vector t:

(12) M=R(S−t)

(13) $$R=\left[\begin{array}{ccc} r_{11} & r_{12} & r_{13}\\ r_{21} & r_{22} & r_{23}\\ r_{31} & r_{32} & r_{33} \end{array}\right]=\left[\begin{array}{c} -0.821028828621\;-0.570886731148\;0.0\;\\ \;0.570886731148\;-0.821028828621\;0.0\\ \;0.000000000000\;0.000000000000\;1.0\; \end{array}\right]$$

(14) $$t=\left[\begin{array}{c} T_{X}\\ T_{Y}\\ T_{Z} \end{array}\;\right]=\left[\begin{array}{c} 9.172649383545\\ 10.196824073792\\ 0.000000000000 \end{array}\;\right]$$

where, S and ℳ represent a source cloud and the target cloud or model, respectively.

Coarse matching was conducted using the 4-Points Congruent Sets (4PCS) algorithm [73]. This technique is rapid, noise-resistance and enables the matching of point clouds with a high number of outliers. As claimed by the authors of this algorithm, cloud pre-filtration and data denoising are not required. The essence of aggressive cloud filtration is the prevention of the loss of significant object elements. Overly aggressive filtration results in the significant loss of high-frequency features, especially in UAV models. The UAV model has significantly fewer high-frequency details. This is manifested by rounded edges of sharp objects, with eliminated small objects. In the case of photogrammetric models, elements smaller than 1.5xGSD (ground sampling distance) are often omitted. The mean GSD for the UAV model is 11 mm; therefore, objects smaller than 16.5 mm will rather be eliminated in the data processing and cloud pre-filtration processes.

**Figure 9:**
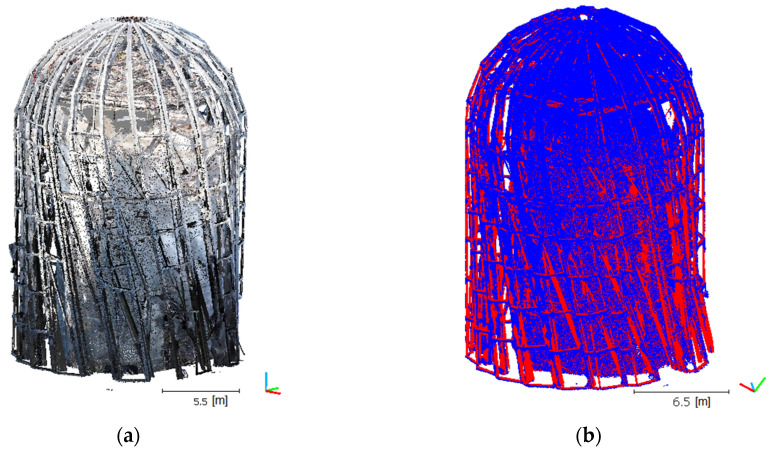
Integrated point clouds using two sources. (**a**) Visualization of the RGB palette points for the UAV cloud and the grayscale for reflection intensity for the UAV and TLS cloud; (**b**) blue—UAV cloud, red—TLS cloud.

The authors of the studies presented in [73], after pre-matching clouds with the 4PCS algorithm, then used precise matching with the ICP algorithm. Moreover, in this case, the ICP algorithm was used within the second stage, where the rotation matrices and translation vectors were also determined. Hence, good cloud pre-matching is important. This stems directly from the ICP algorithm’s principle of operation. In our study, let us assume that S and ℳ represent a source cloud and a target cloud or model, respectively. In this case, the source cloud is the TLS one, while the UAV cloud is considered as the target. Therefore, we are looking for rigid transformation which minimizes the distance between corresponding points in the clouds. The resultant cloud is shown below (Figure 9).

### 2.6. Adaptive Structure Extraction Algorithm

The objective of the extraction of a structural object from an integrated point cloud is the isolation of its stable representatives. These are the point clouds which best represent the geometric structure of the object, regardless of their source, and are noise-independent. In our study, an original automatic and adaptive method involving the extraction of edges from a random point cloud and adaptive thresholding was developed in order to extract the target steel structure. Our method is based on the automatic extraction of edges from a point cloud, as described in [62] and modified using the study [63]. Furthermore, the method by Otsu [78], used in [62], was replaced by adaptive thresholding [79]. This led to the attainment of a new, adaptive and automatic algorithm for the extraction of edges from a point cloud. This algorithm was developed for the extraction of the geometric structure of this particular steel building, as it has a rather complicated shape. However, the algorithm does not exclude universality and its possible application for other purposes. The method is automatic and does not require the provision of any parameters.

The first stage of this algorithm for each point $p_{i}$ of the cloud has a calculated normal vector $\vec{n_{i}}$ for the vicinity of this point that is determined by the *k* nearest neighbouring points. The normal vector $\vec{n_{i}}$ will be equal to the lowest eigenvector corresponding to the lowest eigenvalue of the covariance matrix defined in [80]:

(15) $$C=\frac{1}{k}\;\overset{k}{\underset{i=1}{\sum }}\left(p_{i}-\bar{p}\right)\cdot {\left(p_{i}-\bar{p}\right)}^{T},C\cdot \vec{v_{j}}=\vec{\lambda_{j}}\cdot \vec{v_{j},}\;j\in \left\{0,1,2\right\}\;$$

where *k* is the defined number of neighbours of the query point $p_{i}$, $\bar{p}$ is the centroid for *k* neighbours, $\lambda_{j}$ is the *j* eigenvalue of the covariance matrix, and $\vec{v_{j}}$ is the *j* eigenvector. For a given query point, the $p_{i}$ *k* of the nearest neighbours can be determined through [81].

The neighbours of point $p_{i}$ can be expressed as $V_{i}=\left\{n_{1},n_{2},\ldots ,n_{k}\right\}$; therefore, the centroid $\bar{p_{i\;}}$ for set $V_{i}$ can be calculated from the following formula [63]:

(16) $$\bar{p_{i}}=\frac{1}{\left|V_{i}\right|}\sum_{j=1}^{k}n_{j}$$

The scalar product of vector $\left(\vec{p_{i}-\bar{p_{i}}}\right)$ and the normal vector $\vec{n_{i}}$ in point $p_{i}$ can be expressed as:

(17) $$P_{d\left(i\right)}=\left|\left(\vec{p_{i}-\bar{p_{i}}}\right)\cdot \vec{n_{i}}\right|$$

**Figure 10:**
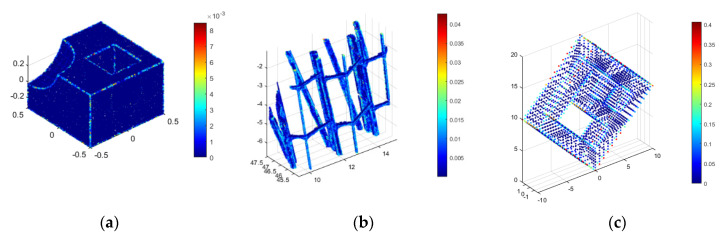
$P_{d}$ values for different tested point clouds: (**a**) sample object 1, (**b**) sample object 2, (**c**) sample object 3.

And will become smaller the more the query point $p_{i}$ will be positioned in the vicinity of the points forming the flat surface [62]. In contrast, the scalar product $P_{d}$ for points located on the edges will adopt the highest values. This method enables the classification of all of the points located on the edge or not. Sample $P_{d}$ values for several cases are shown in Figure 10.

The next stage of the algorithm involves iterative calculations of $P_{d}$ for successive *k* neighbours. In the case in question, it was assumed that *k =* {8, 16, 32 … 128), which gives a total of 16 results for one cloud. If a given edge appears in each iteration for different k values, it can be considered to be a very stable feature. In other words, if a high $P_{d}$ value appears in all of the results at the same point $p_{i}$, it represents the given structure’s stable edge. Thus, if value $P_{d}$ in point $p_{i}$ is equal to or exceeds a certain determined threshold *T*, such a point represents an edge, and conversely, if the value is lower than threshold *T*, it is not treated as an edge. This relationship can be expressed for all iterations as:

(18) $$F\left(i\right)=\{\begin{array}{c} 1\;if\;\overset{ns}{\underset{i=1}{\sum }}P_{d\left(i\right)}\geq T\\ 0\;if\;\overset{ns}{\underset{i=1}{\sum }}P_{d\left(i\right)}<T \end{array}$$

where *T* is defined adaptively, globally for all potential edges, using the adaptive method [79], and *ns* represents the total number of iterations.

In the case of the method in question, the proper determination of the *T* threshold is important. In order to automatically match the value of this threshold, the authors used an adaptive thresholding technique that was discussed in [79]. This algorithm performs its task via two stages. In the first stage, an integral image is calculated based on the source image [82]. In the second stage, the integral image is used to calculate the mean for the value of s×s pixels surrounding each studied image point, followed by a comparison of the pixel values. If the value of the current pixel is *t* percent lower than the calculated mean for its surroundings, then the pixel takes the value 0 (black). Otherwise, it takes the value 1 (white). In the case of this research, *t* = 50%.

## 3. Results and Discussion

### 3.1. Integration Quality Assessment

**Figure 11:**
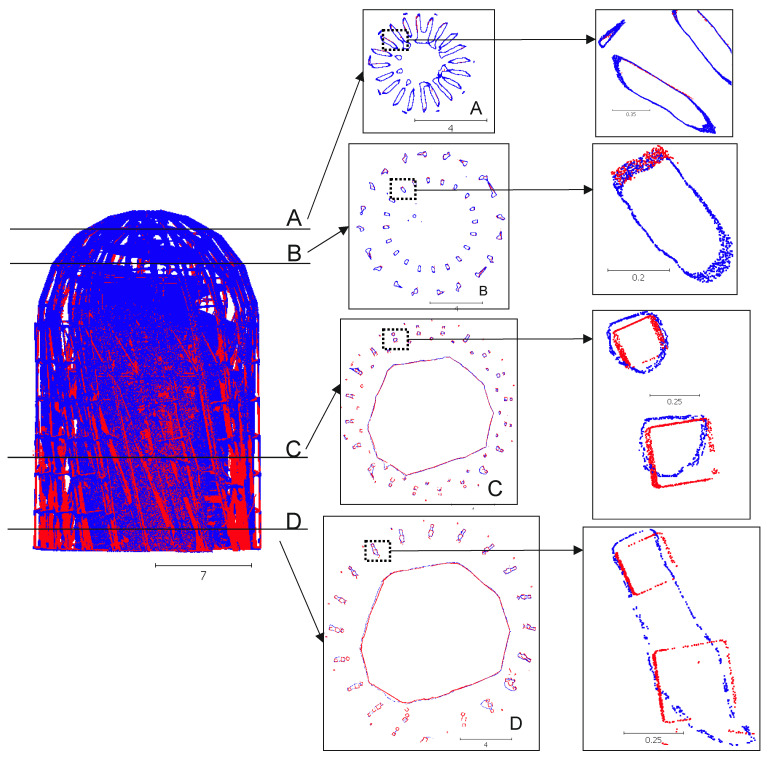
Cross-sections of the integrated point clouds: blue, the UAV cloud; red, the TLS point cloud (values in meters).

**Figure 12:**
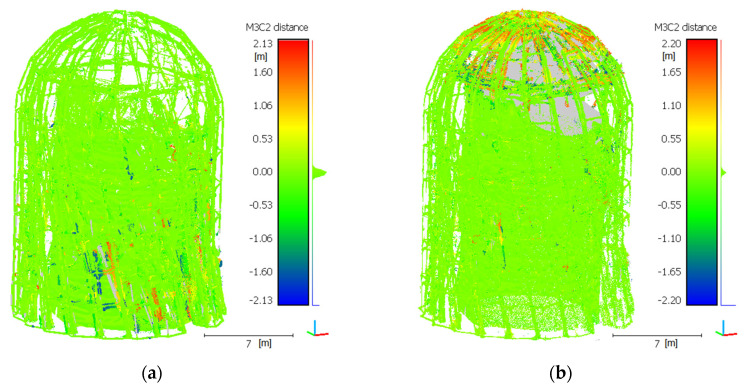
The M3C2 distance for integrated point clouds: (**a**) the distance projected on TLS points; (**b**) the distance projected on UAV points.

The accuracy assessment of the mutual cloud matching after the integration was conducted visually, by developing cross-sections at various levels (Figure 11), and objectively, by using the methods from [83,84]. An M3C2 distance map (Multiscale Model to Model Cloud Comparison) was developed for each point cloud. The results for the processed clouds are shown in Figure 12.

An analysis of the cross-sections based on integrated point clouds on four representative levels (Figure 11) clearly indicates the achieved precision of the integration process and point distribution. Cross-section A, developed at the top of the structure, is characterized by a significant number of UAV points, whereas the TLS points have a trace share in the modelling of the level-A elements. The UAV cloud at level A ensures the required separation between the elements and data continuity within the element cross-section. The TLS cloud, in contrast, does not ensure modelling continuity, and a concentration of TLS points is visible at level B; however, this only takes place on the outer structural elements. The UAV also guarantees element modelling continuity and its separation at this level. Level C exhibits a clear balancing of the modelling continuity for both techniques. The TLS and UAV cloud enables the modelling of elements throughout their entire perimeter; the cross-section is relatively continuous, and the data are available even for internally located structural sections. It is noteworthy that, at the same level, the TLS cloud is a significantly clearer representation of the modelled element, and its shape is precisely reflected. This same element from a UAV cloud is clearly rounded, and its shape is not so sharp. The differences in the distance at this level amount to several millimetres (a maximum of 5 mm) and result from the nature of the very technique of point cloud acquisition and the UAV flight plan. No peripheral flights were detected at this level. In the case of level D, the separation ability of the UAV technique is significantly lower, yet it maintains continuity, although incorrect. The UAV cloud at this level does not enable the modelling of smooth elements in close proximity, because they merge into one shape. In this case, the TLS technique enabled the achievement of a clear structural model, similar to level C.

**Figure 13:**
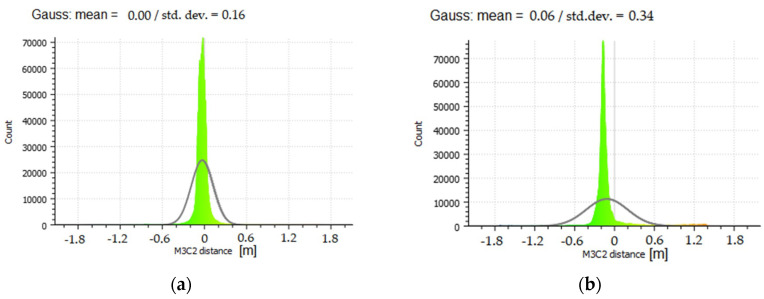
The M3C2 distance for the integrated point clouds: (**a**) the distance projected onto TLS points; (**b**) the distance projected onto UAV points.

When analysing the M3C2 (Figure 13) distance histogram and the normal distribution, it can be concluded that the mean standard deviation is 16 mm, with a mean of 0 for the TLS cloud, which means that this cloud overlaps with the UAV cloud. Because a UAV cloud slightly differs from an actual section course in the bottom part of the structure (as shown by cross-sections C and D in Figure 12), the distance projected onto the UAV cloud indicates a slightly higher standard deviation of 34 mm and a mean of 6 mm. These differences demonstrate that a UAV cloud slightly deviates from an ideal model, especially in the case of the lower parts of the modelled structure. The change in the section shape to a more rounded one can be observed when the number of stations decreases and GSD increases. Conversely, TLS indicates greater shape stability at the expense of the data volume. In the case of the upper structure sections, the TLS cloud (cross-sections A and B in Figure 12) does not map the shape, or maps it very poorly; however, despite the lack of data, the shape is geometrically very correct.

### 3.2. Structure Extraction

The operation of the developed structure edge detection algorithm was validated in two stages. In the first, the algorithm was tested on a source cloud fragment. It involved subjecting the cloud fragment to data reduction, which meant the reduction of the cloud density. The second stage involved testing the operation of the algorithm using the entire source cloud (a fully integrated TLS and UAV point cloud). 

**Figure 14:**
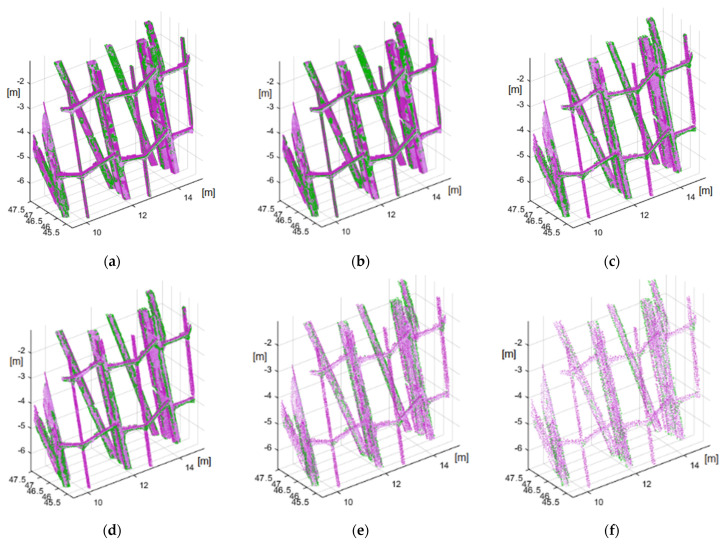
Results for a set of reduced data: (**a**) no reduction, (**b**) 0.5 mm, (**c**) 1 mm, (**d**) 3 mm, (**e**) 5 mm, (**f**) 7 mm (magenta: source points; green: edges detected).

The structural extraction was validated in the first stage on a test set, i.e., a representative fragment of a steel structure. The structure contains fragments of a vertical supporting beam and thinner horizontal supports. Five data sets—such that the minimum distances between the point clouds were 0.5 mm, 1 mm, 3 mm, 5 mm and 7 mm—were developed in order to determine the ability of the algorithm to extract structures and the minimum density of the source cloud. These sets were subsequently subjected to the operation of the developed method, and the results are shown in Figure 14. The source cloud points from a given data set are marked in magenta, and the points of the detected edges are marked in green. 

**Figure 15:**
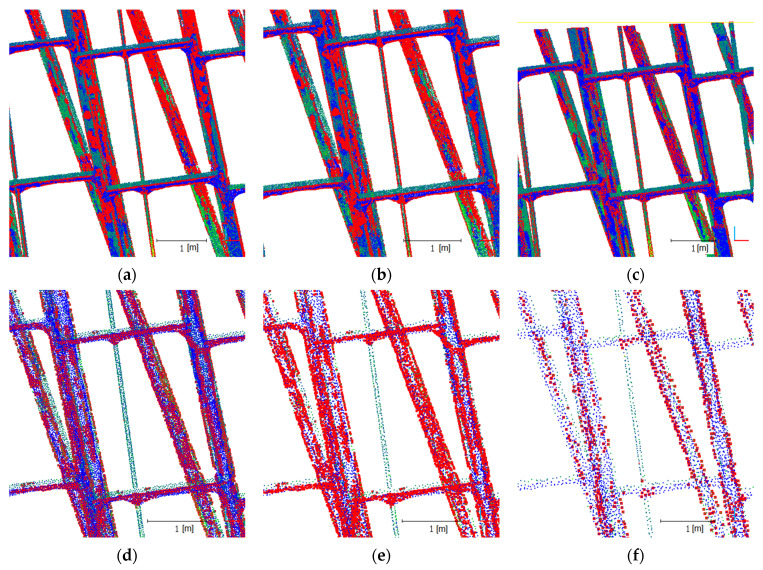
Structure extraction results by the source of the point origin: (**a**) no reduction, (**b**) 0.5 mm, (**c**) 1 mm, (**d**) 3 mm, (**e**) 5 mm, (**f**) 7 mm (green: TLS points; blue: UAV points; red: edges detected).

The results analysis indicated that the developed algorithm extracts structure edges. In the case of a source cloud (not subjected to reduction) (Figure 14a), all of the sharp edges were indicated correctly. These sharp edges originated primarily from laser scanning, and were especially apparent on the horizontal reinforcement beams. UAV points form slightly smoother edges, and point islands appear on some flat surfaces of vertical sections, which are detected as edges. Such a phenomenon occurs at a high density of an irregular point cloud, and is clearly minimized when the distance between cloud points is lower than 3 mm (Figure 14d). The correct edge detection is the case with clouds where the minimum distance between points is 1–3 mm. In the case of these clouds, the edges of vertical beams and of thinner strengthening elements are clearly marked. No loss of data concerning the studied structure is indicated for this density. Further reduction (7 mm) causes the edges of horizontal thinner elements to no longer be detected, with consequent visible loss of data. The described phenomenon occurs for the proposed number of iterations (16) and the highest number of *k* = 128. Because the integrated source point cloud exhibits a very high density, the scale level number (16) planned herein might be insufficient. A larger span of the *k* scale can be used for a higher density, at a clear expense of computing speed loss. However, it should be noted that the nature of the integrated point cloud is not uniform. The cloud originates from two sources. The structure has slightly rounded section edges, such that, with high cloud density, such a potential edge is a rounded section element. In other words, the algorithm is so sensitive that it detects even the smallest edges at a high density, especially on an uneven surface. This unexpected property can sometimes be a great advantage when detecting cracks in particular; however, this was not the goal in this case. Additionally, these surface irregularities originate from the type of applied point cloud acquisition technology, and are notably visible in the case of a UAV cloud. A close-up of this phenomenon is shown in Figure 15. This figure shows clouds divided into UAV (blue), TLS (green) points and detected edge points (red).

**Figure 16:**
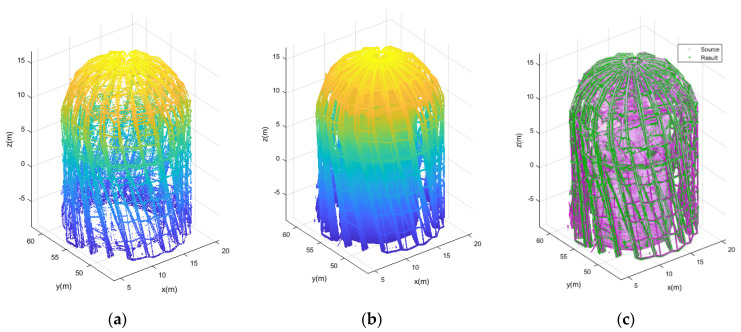
Structure detection results: (**a**) detected edges (results), (**b**) source cloud, (**c**) composite view (source point cloud in magenta, edges (results) in green).

Obtaining the optimal point cloud density enabled us to carry out the final computations for the entire object. The results are shown in Figure 16. The detected edges are shown in the left view and constitute characteristic elements of a steel spatial structure. In the middle is the view of the structures with the source cloud reduced to a value of 3 mm. On the right, we see a composite view, with two clouds representing the source and the detected edges in green, and the baseline cloud in magenta.

The analysis of the ultimate elaboration shows that the essential structural elements have been preserved. The algorithm was very correct in isolating all of the edges of the structural elements and connections. Moreover, the peak rosette has been correctly depicted on the detected edges. Overall, the detected elements enable the conduction of a proper comparative assessment of the steel structure. Such an appraisal is the outcome of comparing the design data and the data acquired as a result of measuring the actual structure. 

## 4. Conclusions

This study shows a comprehensive approach to the issue of processing spatial measurement data using modern techniques. The measured building was a steel structure subjected to verification. The structural verification in the course of construction involved the comparison of its current shape with the design shape. Measurements using terrestrial laser scanning and low-level photogrammetry were conducted for this purpose. Because terrestrial laser scanning was unable to cover the entire structure of the building, its upper part was mapped using data from a UAV. This vehicle was used to reach the peak rosette crowning the building, where it took imagery that was then applied for the construction of a point cloud that was then integrated with the cloud obtained on the basis of the laser scanning. 

This article thoroughly presents the process of the acquisition of measurement data from various sources, as well as their integration and geometric structure extraction. The entire process involved a separate and independent filtration of both point clouds. It also involved the reduction of noise, the number of outliers and the elements of the structure’s surroundings. This filtration was followed by balancing the cloud density and integrating both point clouds. The resulting integrated point cloud enabled an objective presentation of the current geometric state of the building. Because both applied technologies have very broad reality visualization abilities, the reconstructed building had many additional elements that were unnecessary in assessing the geometry of the steel structure itself. Furthermore, the integrated cloud had over 40 million points, which is a maximum reflection of the actual state, but also significantly hinders work in engineering software (a cloud for model assessment and comparison should be smaller than one million points). However, simple data reduction also significantly reduces the important elements of the structure itself. Therefore, such a solution was not considered. In order to extract structurally significant building elements, a new and adaptive algorithm for the extraction of edges from a random point cloud was developed, tested and adopted for the whole process.

The developed adaptive algorithm was based on previously presented studies, but was significantly modified. This algorithm was developed for the extraction of the geometric structure of this particular steel building, as it has a rather complicated shape. However, the algorithm does not exclude universality and its possible application for other purposes. The method is automatic, and does not require the provision of any additional parameters. The applied adaptive thresholding technique enables the algorithm’s operation without specifying the threshold value, thus greatly facilitating the structural extraction process. The developed algorithm correctly detects building element structures based upon the detection of their edges. The object edges were correctly extracted from the integrated cloud, for a minimum point-to-point distance of 1–3 mm. The further reduction of the data for distances between cloud points above 7 mm results in the edges of horizontal thin elements no longer being found, and a visible loss of data.

In contrast to the studies quoted herein, the algorithm was developed and tested by means of actual measurement data. Moreover, data from actual measurements were used to assess the operation. This additionally increases the value of the presented solution. This proves that the adaptive part of the algorithm correctly operates on real data that, in practice, is burdened with irregular noise, processing errors and imperfect shapes. The presented algorithm works for any kind of point cloud. As it was stated above, the point clouds were integrated for the completeness of the data.

One more feature of the developed method was discovered in the course of the study. This, we feel, will be of major importance in the future. In the case of very dense point clouds (a dozen or so points per mm^2^), the algorithm detects even the smallest edges and surface irregularities. This unexpected property could be of great advantage when conducting laser scanning aimed at the detection of microcracking in buildings or other structures.

In order to enable readers to conduct their study and apply the developed algorithm for their own work, we have made the Matlab source code and the developed script available.

## Figures and Tables

**Table 1:** Accuracy-related data of the developed photogrammetric model.

Series	Distance to Object	Ground Resolution	Reprojection Error
1	1–15 m	11 mm/pix	0.71 pix
2	1–15 m	2.4 mm/pix	0.77 pix
	Camera locations and error estimates (mean)		
	X error (m)	Y error (m)	Z error (m)
1	0.00127	0.00137	0.00128
2	0.00082	0.00084	0.00092

**Table 2:** TLS technical data—Leica P30.

Technical Data	Leica P 30
Measurement speed:	Up to 1 MM points per second
Range accuracy:	1.2 mm + 10 ppm over the entire range
Angular accuracy:	8″ horizontally; 8″ vertically
3D position accuracy:	3 mm at 50 m; 6 mm at 100 m
Laser wave length:	1550 nm (invisible)/658 (visible)
Distance noise:	0.4 mm RMS at 10 m 0.5 mm RMS at 50 m
Horizontal field of view:	360°
Vertical field of view:	270°

**Table 3:** Station transformation parameters during stocktaking work involving a steel engineering structure.

Name	PX	PY	PZ	Roll	Pitch	Yaw	Scale	PKT
Stan1	0.000	0.000	0.000	0.000	0.000	0.000	0.0	188
Stan2	0.000	0.000	0.000	0.000	0.011	0.000	0.0	293
Stan3	0.001	0.000	0.003	0.007	−0.015	0.005	0.0	364
Stan4	0.001	0.001	0.004	0.011	−0.015	0.005	0.0	428
Stan5	0.001	0.001	0.007	0.002	0.024	0.005	0.0	330
Stan6	0.000	0.001	0.008	0.017	−0.003	0.001	0.0	359
Stan7	−0.001	0.000	0.011	0.015	−0.021	0.002	0.0	306
Stan8	−0.002	0.000	0.008	0.025	−0.009	0.009	0.0	238
Stan9	−0.001	−0.001	0.007	0.014	0.017	0.006	0.0	304
Stan10	−0.001	−0.001	0.003	0.005	0.012	0.003	0.0	228
Stan11	−0.001	−0.001	0.005	−0.003	0.005	0.002	0.0	245
Stan12	−0.001	−0.002	0.002	−0.006	−0.018	−0.011	0.0	142
Stan13	0.000	−0.002	0.003	0.001	0.003	0.005	0.0	138
Stan14	−0.001	−0.001	0.006	−0.009	−0.008	0.004	0.0	311
Stan15	−0.004	−0.001	0.004	−0.015	−0.003	−0.004	0.0	32
Stan16	−0.009	0.000	0.009	0.001	−0.024	0.010	0.0	536
Stan17	−0.010	0.000	0.009	−0.002	-0.025	0.014	0.0	539

**Table 4:** Number of points in the individual clouds, after each filtration stage.

Filtration Phase	UAV Point Cloud	TLS Point Cloud
Initial	182,505,086	103,680,397
Noise filter	92,214,210	60,791,121
CSF	81,005,411	37,192,129
Manual cleaning	69,029,458	24,033,077
Reduction	24,160,311	24,033,077
SOR	18,806,444	23,875,659

## Data Availability

The sample data and Matlab code for method presented in this study are openly available in repository MOST Wiedzy (https://mostwiedzy.pl/en/) at doi:10.34808/szar-a523.
